# LEVERAGING CRITICAL RURAL GERONTOLOGY TO IMPROVE RURAL GERONTOLOGICAL HEALTH

**DOI:** 10.1093/geroni/igz038.1477

**Published:** 2019-11-08

**Authors:** Laura Poulin, Neil Hanlon

**Affiliations:** 1 Trent University, Ontario, Peterborough, Canada; 2 University of Northern British Columbia, Prince George, British Columbia, Canada

## Abstract

A critical approach in rural gerontology has led to a better understanding of the complex interplay between older adults unique aging experiences and the multidimensional and dynamic communities in which they live. The evolution of critical rural gerontology will be explored, outlining why a similar approach is needed in rural gerontological health. In particular, rural gerontological health literature must expand beyond a deficit focus that homogenizes older adult health experiences and recognize the complexities of negotiating older adult health within multidimensional rural spaces. Inherent in this approach is recognizing the intersectionality of older adult health as well as the need to study rural gerontological health as an experience enhanced and inhibited by interactions within and across formal health services, informal social services and informal care. This approach will contribute to innovations in policy and practice addressing the burgeoning interest of how to effectively care for older adults in rural settings.

